# Retraction Note: The global distribution of acute unintentional pesticide poisoning: estimations based on a systematic review

**DOI:** 10.1186/s12889-024-20318-x

**Published:** 2024-10-09

**Authors:** Wolfgang Boedeker, Meriel Watts, Peter Clausing, Emily Marquez

**Affiliations:** 1 PAN Germany, Nernstweg 32, 22765 Hamburg, Germany; 2 PAN Asia Pacific, P.O. Box 1170, 10850 Penang, Malaysia; 3 PAN North America, 2029 University Ave., Suite 200, Berkeley, CA 94704 USA

**Retraction Note: BMC Public Health (2020) 20:1875**

10.1186/s12889-020-09939-0

The Editor has retracted this article because concerns were raised about the use of ‘ever’ prevalence of pesticide poisoning to represent annual frequency in the extrapolations by a reader and by Dunn et al. [1]. Expert assessment has confirmed the validity of this concern and also concluded that the assumption of annual exposure for countries where the time frame is not reported is unreliable. The Editor therefore no longer has confidence in the results and conclusions presented.

All authors disagree with this retraction.
